# Protein supplementation improves lean body mass in physically active older adults: a randomized placebo‐controlled trial

**DOI:** 10.1002/jcsm.12394

**Published:** 2019-03-07

**Authors:** Dominique S.M. ten Haaf, Thijs M.H. Eijsvogels, Coen C.W.G. Bongers, Astrid M.H. Horstman, Silvie Timmers, Lisette C.P.G.M. de Groot, Maria T.E. Hopman

**Affiliations:** ^1^ Department of Physiology, Radboud Institute for Health Sciences Radboud University Medical Center P.O. Box 9101 Nijmegen The Netherlands; ^2^ FrieslandCampina Amersfoort The Netherlands; ^3^ Human and Animal Physiology Wageningen University Wageningen The Netherlands; ^4^ Division of Human Nutrition and Health Wageningen University Wageningen The Netherlands

**Keywords:** Elderly, Protein, Body composition, Muscle, Randomized clinical trial

## Abstract

**Background:**

An inadequate protein intake may offset the muscle protein synthetic response after physical activity, reducing the possible benefits of an active lifestyle for muscle mass. We examined the effects of 12 weeks of daily protein supplementation on lean body mass, muscle strength, and physical performance in physically active older adults with a low habitual protein intake (<1.0 g/kg/day).

**Methods:**

A randomized double‐blinded controlled trial was performed among 116 physically active older adults [age 69 (interquartile range: 67–73) years, 82% male] who were training for a 4 day walking event of 30, 40, or 50 km/day. Participants were randomly allocated to either 31 g of milk protein or iso‐caloric placebo supplementation for 12 weeks. Body composition (dual‐energy X‐ray absorptiometry), strength (isometric leg extension and grip strength), quadriceps contractile function, and physical performance [Short Physical Performance Battery, Timed Up‐and‐Go test, and cardiorespiratory fitness (Åstrand–Rhyming submaximal exercise test)] were measured at baseline and after 12 weeks. We assessed vitamin D status and markers of muscle damage and renal function in blood and urine samples before and after intervention.

**Results:**

A larger increase in relative lean body mass was observed in the protein vs. placebo group (∆0.93 ± 1.22% vs. ∆0.44 ± 1.40%, *P*_Interaction_ *=* 0.046). Absolute and relative fat mass decreased more in the protein group than in the placebo group (∆−0.90 ± 1.22 kg vs. ∆−0.31 ± 1.28 kg, *P*_Interaction_ *=* 0.013 and ∆−0.92 ± 1.19% vs. ∆−0.39 ± 1.36%, *P*_Interaction_ *=* 0.029, respectively). Strength and contractile function did not change in both groups. Gait speed, chair‐rise ability, Timed Up‐and‐Go, and cardiorespiratory fitness improved in both groups (*P* < 0.001), but no between‐group differences were observed. Serum urea increased in the protein group, whereas no changes were observed in the placebo group (*P*_Interaction_ *<* 0.001). No between‐group differences were observed for vitamin D status, muscle damage, and renal function markers.

**Conclusions:**

In physically active older adults with relatively low habitual dietary protein consumption, an improvement in physical performance, an increase in lean body mass, and a decrease in fat mass were observed after walking exercise training. A larger increase in relative lean body mass and larger reduction in fat mass were observed in participants receiving 12 weeks of daily protein supplementation compared with controls, whereas this was not accompanied by differences in improvements between groups in muscle strength and physical performance.

## Introduction

A physically active lifestyle attenuates the age‐related loss of muscle mass (i.e. sarcopenia) and associated decrements of muscle function^1^, ^2^ by increased muscle protein synthesis rates after exercise but also due to preservation of skeletal muscle sensitivity to dietary amino acids and suppressing the catabolic inflammatory cytokines in the muscle.^3^, ^4^, ^5^ Sufficient protein intake is another vital component to maintain and regain muscle mass.^6^, ^7^, ^8^ Current recommendations for adults advice 0.8 g/kg/day.^9^ However, the PROT‐AGE study group suggested that older adults above 65 years of age should consume 1.0–1.2 g/kg/day to compensate for the attenuated capacity of protein utilization in the aging muscles.^6^ For physically active older adults, their recommendation is even higher, i.e. ≥1.2 g/kg/day in order to comply with the synergistic effects of exercise and protein intake on muscle protein synthesis.^6^ It has previously been shown that more than 50% of physically active older adults has a protein intake below 1.2 g/kg/day.^10^ This observation suggests that physically active older adults may not consume enough protein to be utilized for the exercise‐induced improved muscle protein synthetic response and, thus, to prevent age‐related muscle mass loss.

Therefore, we assessed the effects of 12 weeks of daily protein supplementation on lean body mass, muscle strength, and physical performance in physically active older adults with a low habitual protein intake. We hypothesized that protein supplementation in physically active older adults would induce beneficial effects on lean body mass, muscle strength, and physical performance, while no effects were expected in the control group receiving an iso‐caloric placebo.

## Methods

### Participants

Participants were recruited between 16 March 2017 and 12 April 2017 via the Nijmegen Exercise Study database (Study‐ID: NL36743.091.11^11^) and social media. Interested men and women of at least 65 years old were included if they (i) had a habitual protein intake ≤1.0 g/kg/day based on a 123 item online food frequency questionnaire^12^ calculated using the Dutch Food composition database of 2010,^13^ (ii) were registered and in training for the 2017 Nijmegen Four Days Marches [an annual 4 day walking event (30, 40, or 50 km/day) in the Netherlands; https://www.4daagse.nl/en], and (iii) were able to understand and perform the study procedures. Exclusion criteria for participation in the study were type 1 or type 2 diabetes mellitus (non‐fasted state >11 mmol/L), allergic or sensitive for milk proteins or lactose intolerant, Chronic Obstructive Pulmonary Disease (COPD), cancer, renal insufficiency [estimated glomerular filtration rate (eGFR) < 30 mL/min/1.73 m^−1^], intestinal diseases that may influence the uptake of protein, use of statins, and involved in a heavy resistance type exercise programme. All participants signed an informed consent form prior to any experimental procedure. The study conformed to the principles of the Declaration of Helsinki and was approved by a Medical Ethical committee, the Independent Review Board Nijmegen (Study‐ID: NL60137.072.16). This trial was registered at www.trialregister.nl as NTR6488.

### Design

In a double‐blind, controlled intervention study, a total of 116 eligible participants were randomly allocated to either the protein‐supplemented or the placebo‐supplemented group. An independent researcher randomized the study participants by means of computer‐generated random numbers with a block size of 10 in a 1:1 ratio. Before and after 12 weeks of supplementation, anthropometrics, dual‐energy X‐ray absorptiometry (DXA), strength measurements (maximal isometric leg extension and handgrip strength), and physical performance measurements [Short Physical Performance Battery (SPPB), Timed Up‐and‐Go (TUG), and the Åstrand–Rhyming submaximal exercise test] were performed. Additional muscle function measurements were performed in a subgroup of 30 participants of the protein group and 30 participants of the placebo group. Blood samples, dietary intake (24 h recall), and physical activity (Short Questionnaire to Assess Health enhancing physical activity) data were collected from all participants. In addition, participants were invited to complete an online diary every week, reporting their daily supplement intake and training kilometres (walking) for the Nijmegen Four Days Marches.

### Protein intervention

Participants were asked to consume either a 250 mL protein supplement or a 250 mL iso‐caloric placebo drink, twice a day. Two packages of the protein supplement (500 mL) contained in total 36.8 g milk protein concentrate (MPC 80) with 31 g protein, 1.1 g fat, and 14.5 g lactose (carbohydrates), whereas 500 mL of the placebo supplement contained 1.1 g protein, 5.2 g fat, and 36 g of carbohydrates (FrieslandCampina Consumer Products Europe, Wageningen, the Netherlands). Protein and placebo supplements were provided in ready‐to‐drink non‐transparent packages of 250 mL and were vanilla flavoured to mask contents. Participants were asked to consume one beverage during breakfast and one beverage within 30 min after exercise (e.g. walking). On non‐exercising days, participants were instructed to consume the second beverage during lunch. Participants were asked to report their daily supplement intake every week. Compliance was calculated by dividing the number of used supplements by the total supplements and multiplied by 100. Adverse events were documented.

### Measurements

#### Body composition

Height and weight (Seca 888 scale, Hamburg, Germany) were measured and used to calculate the body mass index. Total and regional lean body mass and fat mass of the participants were measured by DXA (Lunar Prodigy Advance DXA; GE Healthcare, Madison, WI, USA). The DXA scans were performed with dual‐energy beam (0.03 mrem) and a scan time of approximately 10 min.

#### Handgrip strength

Handgrip strength of the dominant hand was measured with a hydraulic, analogue handheld dynamometer (Jamar®, Jackson, MI, USA). For every participant, the dynamometer was adjusted to their hand size. The participants were seated in a chair without arm rests with the elbow flexed in a 90° angle position and were asked to shortly maximally squeeze the handgrip instrument three times with 1 min rest between measurements. The maximum strength in kilograms was used for analysis.

#### Quadriceps strength and contractile function

Additional validated muscle characteristic measurements^14^ were performed in a subgroup of 30 participants of the protein group and 30 participants of the placebo group. Muscle strength was measured by performing three to six isometric maximal voluntary contractions (MVCs) of the dominant quadriceps femoris muscle for approximately 3 s.^15^ The force signal was amplified (strain indicator type CA660, Peekel Instruments, Rotterdam, the Netherlands), digitized (1000 Hz), and stored. The highest MVC was expressed absolute and relative to body weight. Electrically stimulated quadriceps muscle contractions were obtained at 40% of the MVC with 1 s 50 Hz electrical impulses generated by a direct‐current high‐voltage stimulator (DS7A, Digitimer Ltd, Hertfordshire, UK), through two surface electrodes on the distal and proximal part of the anterior thigh (Electro‐Medical Supplies, Greenham Ltd, Wantage, Oxfordshire, UK) to assess voluntary muscle strength, function, and fatigue.^15^ A force–frequency relationship of only the valid measurements that were not limited by technical constraints was obtained through peak force generation upon five 1 s stimulation frequencies (1, 10, 30, 50, and 100 Hz, respectively). Contraction and relaxation rates were calculated as indices of muscle speed of the average of 1, 30, 50, and 100 Hz impulse; normalized maximal rate of force rise was expressed as the maximal slope of force increment as percentage of peak force,^16^ and early and half relaxation time was defined as the time taken for force to decline from 75% to 50% and from 50% to 25% of the peak force, respectively. Resistance to fatigue was assessed by activating the quadriceps muscle repetitively using 30 Hz bursts with a 1 s duration every 2 s for 2 min. Only the valid muscle fatigue resistance measurements were expressed as a percentage of average force of the last three contractions from the average force of the first three contractions, and the peak force per repetition was analysed.

#### Short Physical Performance Battery

Physical performance was assessed using the SPPB, which is considered a reliable and valid method in older adults.^17^ The SPPB consists of three components for which 0–4 points could be earned: balance, gait speed, and chair‐rise ability. Participants' balance was assessed by examining their ability to stand still for 10 s with their feet side by side, in semi‐tandem and in tandem position. Gait speed was determined by the time necessary to complete a walk of 4 m on their usual gait speed. The chair‐rise ability score was determined by the time necessary to rise out of a chair and sit down five times in a row, without aid of arms. For gait speed and chair‐rise ability, the quickest time out of two attempts was reported. A SPPB total score (0–12 points) was calculated by summing the scores.

#### Timed Up‐and‐Go test

During the TUG test, the participants were instructed to rise from a chair, walk 3 m, turn around, walk back, and sit down again as quickly as possible.^18^, ^19^ The time was reported after one trial run.

#### Åstrand–Rhyming test

To evaluate cardiorespiratory fitness, participants performed the Åstrand–Rhyming submaximal exercise test on a stationary bicycle. The test was performed on a mechanically braked cycle ergometer (Corival model, Lode Holding Company BV, the Netherlands), and heart rate was measured with a Polar (Polar Electro, RS400 and RS800 model, Kempele, Finland). The maximal volume of oxygen consumption (VO_2_max) was estimated by applying the work rate and mean heart rate of the 5th and 6th minute to the Åstrand normogram, with correction for weight and age.^20^, ^21^

#### Physical activity

Habitual physical activity was assessed at baseline by the Short Questionnaire to Assess Health enhancing physical activity questionnaire, which is considered a valid and reliable method in older adults.^22^ This self‐administered questionnaire estimates habitual level of physical activity during a normal week over the past month, with questions about the type, duration, and frequency of activities. Total physical activity and exercise‐specific activities were calculated in metabolic equivalent of task hours per day by multiplying the exercise time in hours with the accompanying metabolic equivalent of task score of the activity.^23^ Moreover, participants reported their weekly walking exercise (in kilometres) as a training for the Nijmegen Four Days Marches.

#### Dietary intake

Daily dietary intake was assessed using a repeated 24 h recall, which is a validated method to assess the amount and distribution of protein intake.^24^ Two recall days were randomized over the week with the restriction that no participant was assigned to two identical week days or two weekend days. The 24 h recall was performed face to face or by phone by trained dieticians and coded by the same dieticians into the web‐based program Compl‐eat, which calculated the dietary intake using the Dutch Food Composition Database of 2016.^25^ The mean of the two recorded days represented the daily dietary intake.

#### Blood samples

Non‐fasted venous blood was drawn from the antecubital vein before and after the supplementation period, and serum and lithium heparin samples were stored at −80°C until analysis. Non‐fasting glucose and creatinine levels were assessed to calculate eGFR and were analysed at baseline to exclude participants suffering from insulin resistance, type II diabetes, and renal insufficiency. To check protein intake and renal function before and after the supplementation period, we assessed urea, creatinine, and albumin concentrations. Moreover, we assessed creatine kinase to identify if muscle damage occurred.^26^ Vitamin D status, C‐reactive protein (CRP), and interleukin (IL)‐6 and IL‐10 were assessed, because of their possible confounding effects on muscle mass.^27^, ^28^ Glucose, creatinine, urea, albumin, creatine kinase, and CRP were measured using Siemens Dimension Vista 1500 (Siemens Healthcare Diagnostics Inc., Tarrytown, New York, USA). Serum 25‐hydroxyvitamin D concentrations were measured using liquid chromatography coupled to tandem mass spectrometry detection (Waters Chromatography B.V., Etten‐Leur, the Netherlands). Serum IL‐6 and IL‐10 concentrations were determined using a multiplex electroluminescence‐based cytokine assay on a MESO QuickPlex SQ120 plate imager (Meso Scale Diagnostics, Rockville, Maryland, USA). Analysis were performed by trained technicians using standard operating procedures, on a single day using the same calibration and set‐up to minimize variation.

#### Urine analysis

Upon arrival in the laboratory, a urine sample (5 mL) was provided by all participants and was frozen and stored at −80°C. After completion of the study baseline and post‐supplementation, albumin and creatinine were determined to assess renal function using Dimension Vista 1500 (Siemens Healthcare Diagnostics Inc.).

### Statistical analysis

Based on a Type I error of 0.025 and a power of 90%, we calculated (G‐power, version 3.1.2, University of Dusseldorf, Germany) that 53 participants per study arm were needed to find an expected difference in changes in quadriceps strength of 5 ± 5 kg and 0.41 ± 0.65 in SPPB score between the protein and placebo group.^29^ To account for potential dropout (~10%), we recruited 58 participants per study arm in our study. Statistical analyses were performed using SPSS 22.0 software (IBM SPSS Statistics for Windows, Version 22.0 IBM Corp., Armonk, NY, USA). A per‐protocol analysis was used including only those participants with a compliance rate of ≥90%. All continuous variables were visually inspected and tested for normality with the Shapiro–Wilk test. Participant characteristics were displayed as mean ± standard deviation or mean ± standard error or median [interquartile range (IQR)] for parametric and non‐parametric continuous variables, respectively, and as number of participants with percentages for categorical variables. Baseline characteristics between groups were compared by means of an independent‐samples *t*‐test or a Mann–Whitney U‐test for parametric or non‐parametric continuous variables, respectively, or with a chi‐square test for categorical variables. Data from before and after the supplementation period were analysed by using repeated‐measures analysis of variance with time as a within‐subjects factor and treatment as a between‐subjects factor. Because no between‐group differences were found at baseline, no variables were added as a confounder in the main analysis. The level of significance was set at *P* < 0.05 (two‐sided).

## Results

### Participants

**Figure 1:**
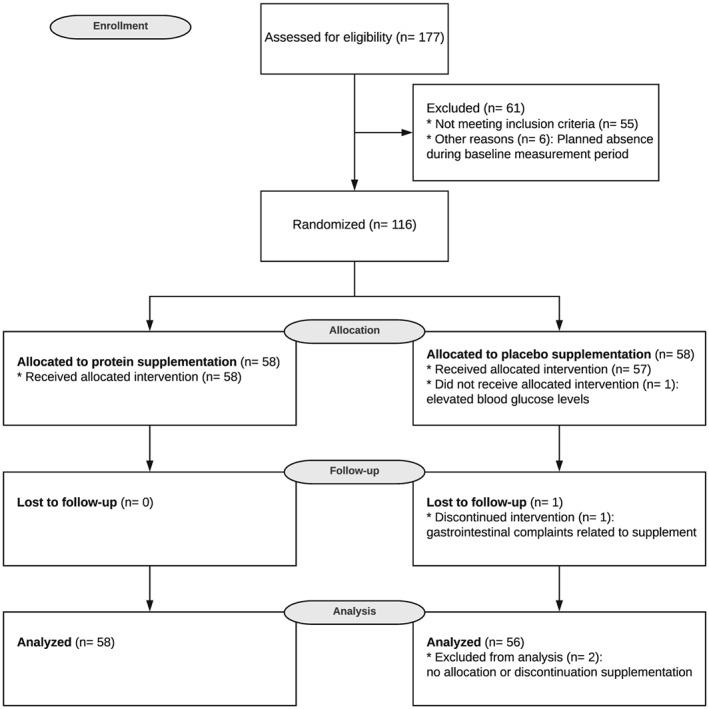
CONSORT flow diagram illustrating the movement of participants through the study, which was conducted between March 2017 and July 2017.

For this study, 177 participants were screened, and 116 participants were included in the study and randomly allocated to the protein or placebo group. One participant had elevated blood glucose levels and was therefore excluded from the study, and another participant dropped out after 2 weeks due to gastrointestinal complaints (*Figure* 1). There were no differences between the protein and placebo groups for any of the baseline characteristics (*Table* 1). Almost all participants were Caucasian, except for one Asian participant in the protein group. Six participants experienced gastrointestinal complaints during the supplementation period (three participants of the protein group and three participants of the placebo group) but did not drop out. There were no serious adverse events reported during the supplementation period. Compliance of supplementation intake was high and did not differ between the protein and placebo groups (96 ± 3% and 95 ± 3%, respectively).

**Table 1:** Baseline characteristics of participants in the protein and placebo group

	Total group	Protein	Placebo	*P*‐value
	*n* = 114	*n* = 58	*n* = 56	
Demographics				
Age (years)	69 (67–73)	69 (67–72)	69 (67–73)	0.82^d^
Men, *n* (%)	93 (82)	47 (81)	46 (82)	0.88^e^
Body composition				
Body weight (kg)	83.1 ± 10.4	84.6 ± 10.2	81.5 ± 10.5	0.11^c^
BMI (kg/m^2^)	26.8 ± 2.6	27.2 ± 2.6	26.3 ± 2.5	0.05^c^
Waist‐to‐hip ratio	0.94 ± 0.08	0.95 ± 0.07	0.94 ± 0.08	0.42^c^
Diet				
Energy intake (kcal)	1944 ± 533	1919 ± 534	1970 ± 536	0.61^c^
Protein intake (g/kg/day)	0.89 ± 0.23	0.86 ± 0.23	0.92 ± 0.24	0.18^c^
Animal protein (%)	61.2 ± 11.1	61.5 ± 11.4	61.1 ± 10.9	0.73^c^
Plant protein (%)	38.8 ± 11.1	38.5 ± 11.4	39.0 ± 10.9	0.73^c^
Protein (en%)	16.0 ± 3.4	16.2 ± 3.1	15.7 ± 3.6	0.44^c^
Fat intake (en%)	35.6 ± 6.7	35.7 ± 7.0	35.5 ± 6.5	0.88^c^
Carbohydrate intake (en%)	42.3 ± 7.3	42.4 ± 8.1	42.1 ± 6.4	0.81^c^
Physical activity				
Total physical activity (METh/week)	117.7 (81.7–173.5)	109.0 (79.1–142.1)	124.0 (87.3–186.1)	0.14^d^
Domestic work activities (METh/week)	26.3 (11.3–45.1)	22.5 (6.3–41.4)	29.5 (15–48.2)	0.14^d^
Commuting activities (METh/week)^a^	0.0 (0.0–0.0)	0.0 (0.0–0.0)	0.0 (0.0–0.0)	0.73^d^
Leisure time activities (METh/week)	53.4 (38.3–73.1)	50.8 (33.0–70.0)	59.3 (39.9–77.9)	0.22^d^
Sports activities (METh/week)	21.0 (3.4–41.2)	21.0 (0.0–39.7)	18.2 (7.8–51.0)	0.74^d^
Blood analysis				
eGFR (mL/min/1.73 m^−1^)	81.2 ± 11.6	79.4 ± 13.5	83.0 ± 9.1	0.11^c^
Non‐fasted glucose (mmol/L)^b^	5.8 ± 1.1	5.7 ± 1.1	5.8 ± 1.2	0.52^c^
25(OH)D (nmol/L)^b^	73.7 ± 27.2	73.7 ± 28.9	73.8 ± 25.6	0.98^c^
CRP (mg/L)	3.9 ± 3.3	4.0 ± 3.8	3.7 ± 2.9	0.66^c^
IL‐6 (pg/mL)	1.02 ± 2.71	0.64 ± 0.44	1.41 ± 3.82	0.13^c^
IL‐10 (pg/mL)	0.305 ± 0.438	0.327 ± 0.446	0.282 ± 0.431	0.58^c^

Data are presented as number (percentage) of participants, mean ± standard deviation, or median (interquartile range). BMI, body mass index; CRP, C‐reactive protein; eGFR, estimated glomerular filtration rate; en%, energy percentage; IL, interleukin; 25(OH)D, 25‐hydroxyvitamin D; MET, metabolic equivalent of task.

a *n =* 22.

b *n =* 113.

c Derived by independent‐samples *t*‐test.

d Derived by Mann–Whitney U‐test.

e Derived by chi‐square test.

### Protein intake

Protein intake was comparable between the protein and placebo groups at baseline (*P =* 0.18), with more than 60% of the proteins derived from animal proteins in both groups (*Table* 1). A significant increase in protein intake (i.e. excluding supplements) was observed over time (*P*_Time_ *=* 0.034), but no differences were observed between groups (*Table* 2). Daily energy and macronutrient intake did not differ between groups at baseline and did not change over time (*Tables* 1 and 2). Taking into account the protein supplements, total protein intake increased in the protein group to 1.29 ± 0.28 g/kg/day during the 12 week supplementation period.

**Table 2:** Changes in habitual dietary intake of participants in the protein and placebo group (disregarding supplements)

	Protein			Placebo			*P*‐value		
	*n* = 58			*n* = 56					
	Pre	Post	Change	Pre	Post	Change	Time	Treatment	Interaction
Energy intake (kcal)	1919 ± 534	1841 ± 456	−77.8 ± 484.5	1970 ± 536	1960 ± 492	−10.4 ± 535.6	0.36	0.30	0.48
Protein intake (g/kg/day)	0.86 ± 0.23	0.92 ± 0.27	0.06 ± 0.27	0.92 ± 0.24	0.97 ± 0.23	0.05 ± 0.28	**0.034**	0.18	0.74
Protein intake at breakfast (g)	11.3 ± 4.8	11.8 ± 7.6	0.5 ± 7.0	12.9 ± 7.7	13.4 ± 7.2	0.3 ± 8.1	0.62	0.17	0.90
Protein intake at lunch (g)	21.7 ± 10.2	20.3 ± 14.5	−3.7 ± 9.3	18.3 ± 7.8	20.7 ± 9.5	2.5 ± 11.6	0.57	0.61	**0.003**
Protein intake at dinner (g)	31.0 ± 10.8	33.8 ± 16.8	2.8 ± 19.5	36.0 ± 14.1	37.4 ± 13.4	1.7 ± 21.3	0.25	**0.014**	0.77
Protein (en%)	16.2 ± 3.1	16.8 ± 3.8	0.6 ± 3.8	15.7 ± 3.6	16.4 ± 3.0	0.7 ± 3.9	0.07	0.41	0.88
Fat (en%)	35.7 ± 7.0	35.5 ± 7.1	−0.2 ± 8.0	35.5 ± 6.5	36.6 ± 6.4	1.1 ± 7.5	0.53	0.64	0.36
Carbohydrate (en%)	42.4 ± 8.1	42.6 ± 7.7	0.1 ± 8.6	42.1 ± 6.4	40.9 ± 7.4	−1.2 ± 8.0	0.51	0.40	0.40

Data are presented as mean ± standard deviation. Bold values indicate *P*‐value <0.05. en%, energy percentage.

### Physical activity

**Figure 2:**
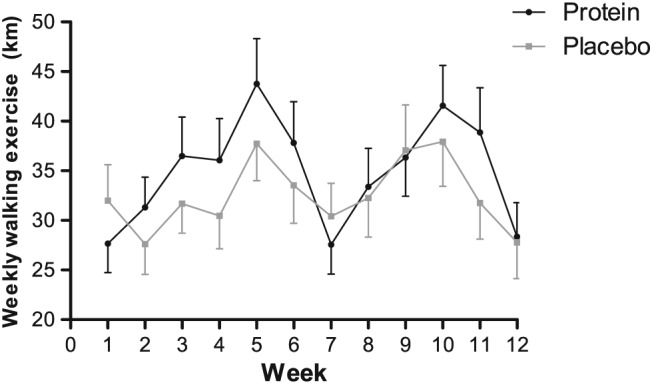
Training walking exercise plotted for every week in kilometres for the protein group, *n =* 58, black lines and for the placebo group, *n =* 56, grey lines. The training kilometres significantly changed over time (*P*_Time_ < 0.001), but no between‐group differences were observed (*P*_Interaction_ = 0.85). Data are presented as mean ± standard error.

Participants of the protein and control group reported a similar physical activity volume at baseline (*Table* 1). All participants performed walking exercise training as a preparation for the Nijmegen Marches. Significant changes over time were observed in training kilometres (*P*_Time_ < 0.001, *Figure* 2), but no between‐group differences were observed (*P*_Interaction_ *=* 0.85). The sum of walking kilometres during the 12 weeks of the study was not different between groups [protein: 391 (IQR: 286–512) km vs. placebo: 338 (IQR: 239–493) km, *P =* 0.31].

### Body composition

**Figure 3:**
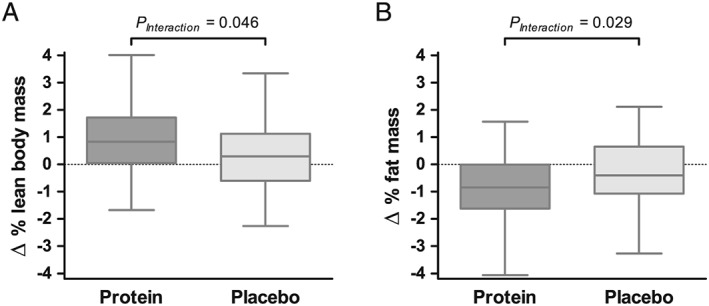
Boxplots showing changes in relative total lean body mass (A) and relative total fat mass (B) in the protein group (dark grey) and placebo group (light grey). There was a significantly larger increase in relative total lean body mass (*P*_Interaction_ = 0.046) and a significantly larger decrease in relative total fat mass in the protein group compared with the placebo group (*P*_Interaction_ = 0.029). Boxplots show the median, upper and lower quartiles, and the maximum and minimum values.

Total body weight decreased borderline significantly more in the protein group compared with the placebo group (*Table* 3). Whole‐body lean mass increased in the protein group as well as in the placebo group following 12 weeks of supplementation (*Table* 3). The protein group had a larger relative increase in whole‐body lean mass than the placebo group (*Table* 3, *Figure* 3). Truncal lean body mass increased significantly more in the protein group compared with the placebo group (*P*_Interaction_ *=* 0.007, *Table* S1). Total body fat mass decreased in both groups but significantly more in the protein group compared with the placebo group (*Table* 3). Furthermore, fat mass/lean body mass ratio was significantly more reduced in the protein group compared with the placebo group (*Table* 3).

**Table 3:** Changes in body composition, strength, physical performance, and blood and urine parameters of participants in the protein and placebo group

	Protein			Placebo			*P*‐value		
	*n* = 58			*n* = 56					
	Pre	Post	Change	Pre	Post	Change	Time	Treatment	Interaction
Body composition									
Body weight (kg)	84.59 ± 10.22	84.00 ± 10.28	−0.59 ± 1.41	81.17 ± 10.33	81.02 ± 10.20	−0.15 ± 1.12	**0.003**	0.10	0.07
Lean body mass (kg)	56.80 ± 7.97	57.34 ± 8.17	0.54 ± 1.13	56.71 ± 9.35	57.02 ± 9.21	0.31 ± 1.03	**<0.001**	0.90	0.27
Lean body mass (%)	66.71 ± 5.93	67.64 ± 5.74	0.93 ± 1.22	68.92 ± 7.08	69.36 ± 7.00	0.44 ± 1.40	**<0.001**	0.11	**0.046**
Fat mass (kg)	25.10 ± 6.28	24.20 ± 6.12	−0.90 ± 1.22	22.20 ± 6.19	21.89 ± 6.24	−0.31 ± 1.28	**<0.001**	**0.026**	**0.013**
Fat mass (%)	29.39 ± 6.25	28.47 ± 6.12	−0.92 ± 1.19	27.11 ± 7.30	26.72 ± 7.22	−0.39 ± 1.36	**<0.001**	0.11	**0.029**
Ratio fat mass/lean body mass	0.43 ± 0.13	0.41 ± 0.12	−0.02 ± 0.03	0.39 ± 0.15	0.38 ± 0.14	−0.01 ± 0.03	**<0.001**	0.17	**0.032**
Strength									
MVC, N^a^	698 ± 180	706 ± 175	7.2 ± 71.6	691 ± 163	683 ± 163	−8.7 ± 63.1	0.94	0.74	0.38
MVC/kg body weight, N^a^	8.2 ± 1.8	8.4 ± 1.7	0.1 ± 0.9	8.1 ± 1.9	8.3 ± 1.8	0.2 ± 1.6	0.31	0.78	0.84
Maximal rate of force rise (%/ms)^b^	1.20 ± 1.13	1.14 ± 0.12	−0.06 ± 0.09	1.21 ± 0.13	1.18 ± 0.14	−0.03 ± 0.11	**0.004**	0.52	0.38
Early relaxation time (ms)^c^	27.3 ± 4.3	27.1 ± 4.3	−0.27 ± 3.05	25.9 ± 3.9	26.2 ± 4.1	0.29 ± 2.7	0.98	0.41	0.58
Half relaxation time (ms)^d^	35.6 ± 7.1	36.7 ± 9.0	1.1 ± 8.5	37.4 ± 5.6	38.9 ± 6.7	1.5 ± 4.5	0.39	0.47	0.87
Fatigue (%)^e^	−30 ± 8	−30 ± 8	−0.6 ± 8.5	−31 ± 10	−30 ± 10	1.1 ± 7.4	0.86	0.85	0.57
Grip strength (kg)	37 ± 8	41 ± 9	0 ± 4	38 ± 10	43 ± 11	1 ± 4	0.12	0.37	0.24
Physical performance									
SPPB total (pt)	12 (11–12)	12 (11–12)	0 (0–1)	12 (11–12)	12 (12–12)	0 (0–0)	0.10	0.41	0.73
Balance (pt)	4 (4–4)	4 (4–4)	0 (0–0)	4 (4–4)	4 (4–4)	0 (0–0)	1.00	0.68	1.00
Gait speed (pt)	4 (4–4)	4 (4–4)	0 (0–0)	4 (4–4)	4 (4–4)	0 (0–0)	—	—	—
Gait speed (s)	3.2 ± 0.3	2.9 ± 0.4	−0.2 ± 0.5	3.2 ± 0.5	3.0 ± 0.4	−0.2 ± 0.4	**<0.001**	0.72	0.95
Chair‐rise (pt)	4 (3–4)	4 (3.8–4)	0 (0–1)	4 (3–4)	4 (4–4)	0 (0–0)	0.07	0.25	0.70
Chair‐rise (s)^f^	10.4 ± 2.2	9.7 ± 2.2	−0.8 ± 2.2	10.2 ± 1.8	9.4 ± 2.3	−0.7 ± 1.9	**<0.001**	0.77	0.86
TUG (s)	6.9 ± 0.9	6.5 ± 0.8	−0.4 ± 0.9	6.9 ± 1.2	6.5 ± 1.1	−0.5 ± 0.6	**<0.001**	0.96	0.50
Estimated VO_2_max (mL/kg/min)^g^	31.1 ± 9.9	34.7 ± 12.1	3.6 ± 7.8	29.5 ± 9.1	32.5 ± 10.6	3.1 ± 6.8	**<0.001**	0.31	0.71
Blood parameters									
25(OH)D (nmol/L)^h^	73.7 ± 28.9	93.4 ± 21.1	20.1 ± 19.9	73.8 ± 25.6	96.0 ± 25.8	21.7 ± 16.3	**0.001**	0.76	0.48
Creatine kinase (U/L)^h^	148.5 ± 77.1	134.5 ± 76.2	−14.0 ± 57.6	138.2 ± 69.6	140.6 ± 79.9	3.5 ± 57.6	0.34	0.91	0.11
Albumin (g/L)^h^	41.3 ± 2.3	41.3 ± 2.3	−0.1 ± 2.1	41.2 ± 2.2	41.6 ± 2.6	0.4 ± 2.1	0.44	0.76	0.20
Creatinine (μmol/L)	80.8 ± 16.3	84.7 ± 17.2	3.9 ± 11.0	77.9 ± 13.1	82.8 ± 13.4	4.9 ± 6.8	**<0.001**	0.37	0.56
eGFR (mL/min/1.73 m^−1^)^i^	79.4 ± 13.5	78.1 ± 13.4	−1.9 ± 9.8	83.0 ± 9.1	78.7 ± 10.1	−4.1 ± 7.0	**<0.001**	0.42	0.19
Urea (mmol/L)^h^	5.9 ± 1.6	8.3 ± 2.3	2.5 ± 1.5	6.1 ± 1.4	6.2 ± 1.2	0.2 ± 1.3	**<0.001**	**0.001**	**<0.001**
Urine parameters									
Albumin/creatinine ratio (mg/mmol)^j^	2.7 ± 4.0	2.2 ± 2.3	−0.5 ± 3.3	2.5 ± 4.6	2.5 ± 4.2	−0.4 ± 4.1	0.18	0.84	0.86

Data are presented as mean ± standard deviation or median (interquartile range). Bold values indicate *P*‐value <0.05. 25(OH)D, 25‐hydroxyvitamin D; eGFR, estimated glomerular filtration rate; MVC, maximal voluntary contraction; SPPB, Short Physical Performance Battery; TUG, Timed Up‐and‐Go; VO_2_max, maximal rate of oxygen consumption.

a *n =* 56.

b *n =* 44.

c *n =* 33.

d *n =* 22.

e *n =* 30.

f *n =* 111.

g Estimated VO_2_max, corrected for age and weight with the Åstrand test (*n =* 112).

h *n =* 113.

i *n =* 109.

j *n =* 111.

### Muscle strength and contractile function

**Figure 4:**
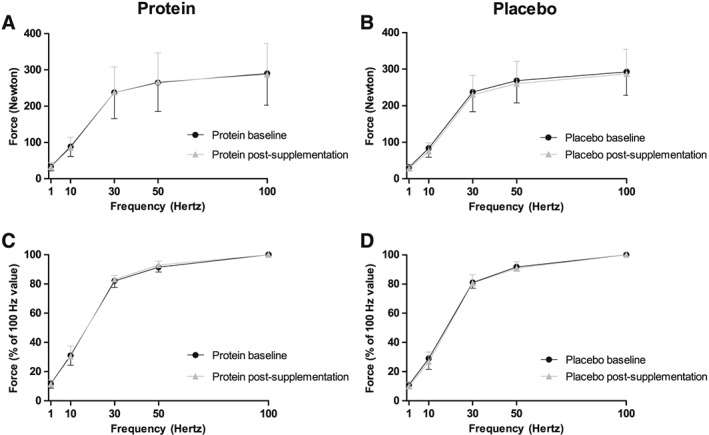
Force responses to different stimulation frequencies (1, 10, 30, 50, and 100 Hz) are given in absolute forces (A and B) and normalized for peak isometric 100 Hz force (relative) (C and D) at baseline and after the supplementation period for the protein group, *n =* 20 (A and C) and for the placebo group, *n =* 24 (B and D). At baseline, the absolute and relative peak forces of the quadriceps were similar between the protein and placebo groups (*P*_Interaction_ *=* 0.75 and *P*_Interaction_ *= 0.75*, respectively). After the supplementation, again no between‐group differences were observed in the absolute and relative quadriceps peak forces (*P*_Interaction_ *=* 0.33 and *P*_Interaction_ *=* 0.20, respectively). Data are presented as mean ± standard deviation.

**Figure 5:**
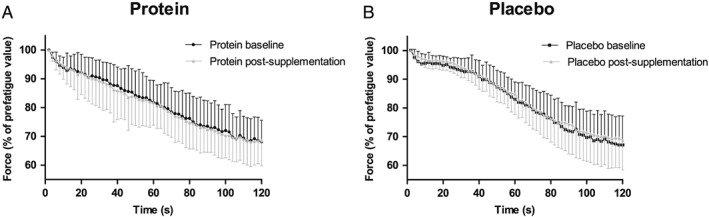
Force responses plotted every second during the fatigue protocol at baseline (t0) and after the supplementation period (t1) for the protein group, *n =* 14 (A) and for the placebo group, *n =* 16 (B). At baseline, the decline in force of the quadriceps was similar between the protein and placebo groups (*P*_Interaction_ *=* 0.17). For both groups, a significant decline in quadriceps force was observed at baseline and after the supplementation (all *P*_Time_ *<* 0.001). After the supplementation, again no between‐group differences were observed in the decline in quadriceps force (*P*_Interaction_ *=* 0.27). Data are presented as mean ± standard deviation.

Handgrip strength was not improved in both groups after the supplementation period (*Table* 3). Subgroup measurements of maximal voluntary quadriceps contraction demonstrated also no changes (*Table* 3). Electrically stimulated quadriceps muscle peak contractions to 1, 10, 30, 50, and 100 Hz for the two groups are shown in *Figure* 4, and no between‐group differences were observed at baseline or over time. Maximal rate of force rise and early and half relaxation time were not different between groups over time (*Table* 3), indicating that no differences occurred in velocity response of the muscle. Muscle fatigue, the significant decline in force of the quadriceps muscle during 2 min of electrical stimulation, did not differ between groups at baseline and after the supplementation period (*Figure* 5). Finally, no changes in resistance to fatigue after 2 min of electrical stimulation were found in both groups (*Table* 3).

### Physical performance

No significant change in total SPPB score was observed in both the protein and placebo group after the intervention (*Table* 3). After 12 weeks, both groups showed faster gait speed (*P*_Time_ *<* 0.001), faster chair‐rise ability (*P*_Time_ *<* 0.001), faster TUG (*P*_Time_ *<* 0.001), and increased estimated VO_2_max (*P*_Time_ *<* 0.001), but no differences between groups were observed in any of the SPPB subscores, TUG, or estimated VO_2_max (*Table* 3).

The effects of 12 weeks of daily protein vs. placebo supplementation on body composition, muscle strength, and physical performance are separately given for men and women in *Table* S2A and S2B.

### Biochemical measures

Renal function (eGFR), glucose levels, and inflammatory markers (CRP, IL‐6, and IL‐10) were similar at baseline (*Table* 1). At baseline, 79% of the protein group and 84% of the placebo group had a serum 25‐hydroxyvitamin D of ≥50 nmol/L. The vitamin D serum levels increased in both groups (*P*_Time_ *=* 0.001), but no between‐group differences were observed (*Table* 3). In both the protein and placebo groups, creatinine concentrations increased, and eGFR decreased after 12 weeks (both *P*_Time_ *<* 0.001), but no between‐group differences were observed (*Table* 3). Serum urea, a breakdown product of protein, increased following 12 weeks of protein supplementation in the protein group, whereas no changes were observed in the placebo group (*Table* 3). No differences were observed between groups for serum creatine kinase, serum albumin, and urinary albumin/urinary creatinine ratio following 12 weeks of supplementation (*Table* 3).

## Discussion

The present randomized double‐blind placebo‐controlled trial revealed novel findings about the benefits of 12 weeks protein supplementation in physically active older adults with a low habitual dietary protein intake. First, we found a larger relative increase in lean body mass and a larger decrease in fat mass in the protein intervention group vs. control group. However, no differences in muscle strength, muscle contractile properties, and physical performance were found over time between groups. These findings suggest that age‐related loss of muscle mass can be delayed with an increased protein intake in physically active older adults who have a relatively low habitual protein intake, while no changes were observed in muscle function.

Twelve weeks of protein supplementation induced a relative increase of whole‐body lean mass by 0.93 ± 1.22% and a concomitant decrease in fat mass in physically active older adults, which was larger than changes observed in the placebo group. These results are in line with previous studies that investigated the benefits of protein supplementation in frail older adults,^30^, ^31^, ^32^ while studies assessing the effect of protein supplementation in community‐dwelling older adults found contradicting results. Whereas some studies in community‐dwelling older adults found improvements of lean body mass with protein supplementation,^33^, ^34^, ^35^ others did not find such beneficial effects.^36^, ^37^ A potential explanation for these discrepant findings may relate to differences in the included participants. We specifically selected physically active older adults with a low habitual protein intake based on the FFQ. It has been shown that regular exercise training stimulates muscle protein synthesis, but the muscle protein balance remains negative in the absence of sufficient protein intake.^38^ Hence, community‐dwelling older adults that are not as active as our participants may not benefit from protein supplementation as there is insufficient stimulus for muscle synthesis. Alternatively, we supplemented our physically active participants with 15 g protein at breakfast and 15 g protein after exercise or at lunch, causing a significant increase in daily protein intake from 0.86 ± 0.23 g/kg/day upon enrolment to 1.29 ± 0.28 g/kg/day at 12 weeks. This level of protein intake aligns with guideline recommendations for physically active older adults^6^ and seemed sufficient to attenuate the age‐induced loss of muscle mass in previous studies.^39^, ^40^, ^41^

The increase in lean body mass and decrease in fat mass were predominantly observed in the trunk. These findings are in alignment with previous studies that revealed an increase in trunk lean body mass following aerobic exercise training, whereas resistance exercise also increased appendicular lean body mass.^42^, ^43^ Our participants of both the protein and placebo groups mainly performed moderate intensity walking exercise, which might explain the trunk‐specific improvements in both groups. The improvements were however significantly larger in the protein group. Various health benefits have been associated with truncal body composition improvements, such as a reduced risk for cardiovascular diseases and metabolic syndrome,^44^ improved postural stability, and consequently a reduced risk for falls,^45^, ^46^ while the maintenance of lean mass of the trunk may only moderately contribute to the mobility of older adults.^47^

We did not find improvements in hand grip strength, nor in quadriceps muscle strength, contractile function and fatigue following protein supplementation. These muscle characteristics all apply to appendicular muscles, while lean body mass mainly increased in the trunk region, which may partly explain the lack of improvements seen in these muscles. Lean body mass improvements are certainly not always accompanied by changes in muscle strength,^39^ as sometimes, the muscular hypertrophy is not induced by myofibrillar hypertrophy but by sarcoplasmic hypertrophy.^48^ The latter consists of growth of the sarcoplasm and non‐contractile proteins, thus not directly contributing to muscular force.^48^ Because no biopsies were performed in our volunteers, the identification of the compartment that accumulates proteins cannot be addressed in this study.

While both groups increased their cardiorespiratory fitness, most likely as a result of the increased walking exercise training kilometres, no between‐group differences were observed. A previous study showed positive effects of protein supplementation on changes in VO_2_max among participants aged 48 ± 7 years.^49^ However, the participants of the treatment group included in that study were untrained and had lower cardiorespiratory fitness scores at baseline compared with the baseline values of estimated VO_2_max of our participants (25.5 ± 4.2 mL/kg/min vs. 31.1 ± 9.9 mL/kg/min, respectively). Untrained participants may benefit more from protein supplementation for improvement of aerobic fitness, than physically active older adults do.^50^

Although physical performance as measured with SPPB and TUG improved in both groups after 12 weeks, most likely as a result of the increased walking exercise training kilometres, protein supplementation had no additional impact on these changes. The beneficial effects of a physically active lifestyle might therefore be more pronounced and overrule the benefits of enhancing the protein intake. A study performed in active older men found no additional effect of protein supplementation above the effect of resistance exercise training^51^ indicating that the effect of exercise is larger than the effect of protein intake.^38^ However, the active older men that were studied had an adequate protein intake (1.14 ± 0.05 g/kg/day) already. The results of our study suggest that improving the protein intake in healthy active elderly with an inadequate habitual protein intake can enlarge the health benefits of an active lifestyle by increasing lean body mass. Moreover, it should be noted that the physically active older adults in our study exhibited already a high level of physical performance at baseline [median: 12 (IQR: 11–12) with 65% of the participants demonstrating the maximum score of 12 points at baseline], and consequently, it was likely that a ceiling effect occurred for most participants. Therefore, SPPB may not be an adequate test in this active group to assess the effect of additional protein supplementation.^52^ In parallel, the TUG test reports in community‐dwelling older adults average scores between 7.9 and 9.0 s,^53^, ^54^ whereas our participants already scored 6.9 ± 0.9 s at baseline, thus creating a small window for improvement. Therefore, we should be cautious with our findings that protein supplementation had no effect on physical performance because our tests used may not have been suitable for such an active population. Alternative tests such as 400 m walk test generally give more information in high‐functioning participants^52^ and are recommended to be incorporated in future studies.

The results of the present study suggest that physically active older adults with a low habitual protein intake could gain almost 1% in lean body mass following 12 weeks of protein supplementation of 31 g/day. The average rate of annual loss of muscle mass in older adults is normally approximately 0.5–1.0%.^40^ Thus, the increase of lean body mass found in the protein group could be translated into saving 1–2 years of muscle mass decline and is therefore of great significance for daily life mobility on the long term. The enhanced protein intake did not seem to affect renal function throughout the supplementation period because no differences in eGFR were observed compared with the placebo group and no changes in urinary albumin/urinary creatinine ratio were seen over time. Therefore, enhancing protein intake is not only effective but also a safe strategy^55^ to attenuate the age‐related loss of muscle mass in physically active older adults.

We performed a double‐blinded randomized placebo‐controlled trial in a large study population with a low dropout rate and high compliance. However, some limitations should be noted. Our physical performance measurements were most likely not sensitive enough to distinguish improvements between both groups of high‐functioning participants. Furthermore, we did not collect 24 h urine in which creatinine could be determined, the gold standard to assess renal function. However, with other parameters such as serum eGFR and urinary albumin/urinary creatinine ratio, we were able to determine that renal function was unaffected by the supplementation. We performed explorative sex‐specific analyses of our data and found that the beneficial effects of protein supplementation on body composition are more pronounced in women than in men. We acknowledge that our study was not powered for these sub‐analyses, but the outcomes suggest that more studies are warranted to assess possible differences between men and women in responses to protein supplementation.

## Conclusions

In physically active older adults with relatively low habitual dietary protein consumption, an improvement in physical performance, an increase in lean body mass, and a decrease in fat mass were observed after walking exercise training. Twelve weeks of protein supplementation resulted in a relative larger increase in lean body mass and a larger decrease in fat mass compared with the placebo group. This was however not accompanied by differences in improvements in muscle strength or physical performance between both groups. The improved body composition shows that protein supplementation enlarges the proposed health benefits of an active lifestyle in physically active older adults, but physical performance could not be improved further in already vital older adults.

## Conflict of interest

None declared.

## Funding

This research is funded by the ‘Topconsortia voor Kennis en Innovatie (TKI's)’ from the ministry of Ministry of Economic Affairs, ‘TKI Agri & Food’, the Netherlands.

## Ethical standards

The authors certify that they comply with the ethical guidelines for authorship and publishing of the *Journal of Cachexia, Sarcopenia, and Muscle*: update 2015.^56^

## Supporting information


**Table S1.** Changes in regional body composition of participants in the protein and placebo group

**Table S2.** (A) Changes in body composition, strength, physical performance, blood and urine parameters of the male participants in the protein and placebo group. (B) Changes in body composition, strength, physical performance, blood and urine parameters of the female participants in the protein and placebo group.
