# Supplementary material for: Protein supplementation improves lean body mass in physically active older adults: a randomized placebo‐controlled trial
Source: J Cachexia Sarcopenia Muscle. 2019 Mar 7;10(2):298–310. doi: 10.1002/jcsm.12394 (PMC6463466; doi:10.1002/jcsm.12394)
Supplement: Supplementary file 1 — Table S1. Changes in regional body composition of participants in the protein and placebo group [file JCSM-10-298-s001.docx]

| **Online Resource 1** Changes in regional body composition of participants in the protein and placebo group | | | | | | | | | | | | | | | | | |
| --- | --- | --- | --- | --- | --- | --- | --- | --- | --- | --- | --- | --- | --- | --- | --- | --- | --- |
|  | **Protein** n=58 | | |  | | | | **Placebo** n=56 | | | | |  | | **P-value** | | |
|  | Pre | Post | Change | |  | | Pre | | Post | | Change |  | | | Time | Treatment | Interaction |
| **Trunk** | | | | | | | | | | | | | | | | | |
| Trunk lean body mass, kg | 27.6 ± 3.7 | 27.9 ± 3.9 | 0.3 ± 0.9 | | | 27.2 ± 4.5 | | | 27.3 ± 4.4 | 0.1 ± 1.0 | | | | **0.038** | | 0.49 | **0.007** |
| Trunk lean body mass, % | 63.1 ± 5.9 | 64.0 ± 5.9 | 0.9 ± 1.5 | | | 65.0 ± 7.3 | | | 65.3 ± 7.2 | 0.3 ± 2.1 | | | | **0.001** | | 0.20 | **0.047** |
| Trunk fat mass, kg | 15.4 ± 4.2 | 14.9 ± 4.2 | -0.5 ± 0.9 | | | 13.7 ± 4.1 | | | 13.7 ± 4.2 | 0.0 ± 1.0 | | | | **0.003** | | 0.06 | **0.011** |
| Trunk fat mass, % | 34.6 ± 6.0 | 33.6 ± 6.0 | -1.0 ± 1.6 | | | 32.5 ± 7.3 | | | 32.3 ± 7.3 | -0.1 ± 2.0 | | | | **0.002** | | 0.17 | **0.017** |
| **Arms** |  |  |  | | |  | | |  |  | | | |  | |  |  |
| Arm lean body mass, kg | 6.0 ± 1.2 | 6.0 ± 1.2 | 0.0 ± 0.4 | | | 6.0 ± 1.4 | | | 6.0 ± 1.3 | 0.0 ± 0.2 | | | | 0.89 | | 0.84 | 0.51 |
| Arm lean body mass, % | 67.9 ± 7.2 | 67.7 ± 6.6 | -0.2 ± 1.9 | | | 69.6 ± 7.6 | | | 69.5 ± 7.3 | -0.1 ± 1.8 | | | | 0.41 | | 0.18 | 0.78 |
| Arm fat mass, kg | 2.4 ± 0.8 | 2.4 ± 0.7 | 0.0 ± 0.3 | | | 2.1 ± 0.6 | | | 2.1 ± 0.6 | 0.0 ± 0.2 | | | | 0.54 | | **0.029** | 0.68 |
| Arm fat mass, % | 27.1 ± 7.8 | 27.3 ± 7.1 | 0.2 ± 2.1 | | | 25.3 ± 8.0 | | | 25.4 ± 7.7 | 0.1 ± 1.8 | | | | 0.47 | | 0.18 | 0.69 |
| **Legs** |  |  |  | | |  | | |  |  | | | |  | |  |  |
| Leg lean body mass, kg | 19.4 ± 3.1 | 19.6 ± 3.1 | 0.2 ± 0.7 | | | 19.6 ± 3.5 | | | 19.7 ± 3.5 | 0.2 ± 0.7 | | | | **0.003** | | 0.85 | 0.51 |
| Leg lean body mass, % | 71.1 ± 8.5 | 72.3 ± 8.3 | 1.2 ± 1.4 | | | 73.6 ± 9.1 | | | 74.6 ± 9.1 | 1.0 ± 1.4 | | | | **<0.001** | | 0.15 | 0.31 |
| Leg fat mass, kg | 6.5 ± 2.6 | 6.2 ± 2.5 | -0.4 ± 0.5 | | | 5.6 ± 2.3 | | | 5.3 ± 2.3 | -0.3 ± 0.4 | | | | **<0.001** | | **0.049** | 0.22 |
| Leg fat mass, % | 23.9 ± 9.0 | 22.7 ± 8.8 | -1.3 ± 1.4 | | | 21.5 ± 9.5 | | | 20.5 ± 9.4 | -1.0 ± 1.4 | | | | **<0.001** | | 0.18 | 0.26 |
| Data are presented as mean ± SD. Bold values indicate p-value < 0.05. | | | | | | | | | | | | | | | | | |
