# Supplementary material for: Protein supplementation improves lean body mass in physically active older adults: a randomized placebo‐controlled trial
Source: J Cachexia Sarcopenia Muscle. 2019 Mar 7;10(2):298–310. doi: 10.1002/jcsm.12394 (PMC6463466; doi:10.1002/jcsm.12394)
Supplement: Supplementary file 2 — Table S2. (A) Changes in body composition, strength, physical performance, blood and urine parameters of the male participants in the protein and placebo group. (B) Changes in body composition, strength, physical performance, blood and urine parameters of the female participants in the protein and placebo group. [file JCSM-10-298-s002.docx]

| **Online Resource 2A** Changes in body composition, strength, physical performance, blood and urine parameters of the male participants in the protein and placebo group | | | | | | | | | | | | | | | | | |
| --- | --- | --- | --- | --- | --- | --- | --- | --- | --- | --- | --- | --- | --- | --- | --- | --- | --- |
|  | **Protein** n=47 | | |  | | | | **Placebo** n=46 | | | | |  | | **P-value** | | |
|  | Pre | Post | Change | |  | | Pre | | Post | | Change |  | | | Time | Treatment | Interaction |
| **Body composition** | | | | | | | | | | | | | | | | | |
| Body weight, kg | 83.40 ± 9.11 | 85.98 ± 9.03 | -0.42 ± 1.41 | | | 84.04 ± 8.65 | | | 83.80 ± 8.62 | -0.25 ± 1.08 | | | | **0.013** | | 0.22 | 0.51 |
| Lean body mass, kg | 59.64 ± 5.43 | 60.18 ± 5.75 | 0.54 ± 1.09 | | | 60.32 ± 5.29 | | | 60.59 ± 5.23 | 0.26 ± 0.95 | | | | **<0.001** | | 0.63 | 0.20 |
| Lean body mass, % | 68.61 ± 4.48 | 69.36 ± 4.57 | 0.75 ± 1.00 | | | 71.07 ± 5.40 | | | 71.49 ± 5.34 | 0.42 ± 1.27 | | | | **<0.001** | | **0.027** | 0.16 |
| Fat mass, kg | 24.06 ± 5.67 | 23.36 ± 5.67 | -0.7 ± 1.08 | | | 21.43 ± 6.20 | | | 21.10 ± 6.26 | -0.34 ± 1.22 | | | | **<0.001** | | **0.050** | 0.13 |
| Fat mass, % | 27.35 ± 4.65 | 26.62 ± 4.76 | -0.74 ± 1.00 | | | 24.84 ± 5.51 | | | 24.47 ± 5.46 | -0.37 ± 1.21 | | | | **<0.001** | | **0.029** | 0.12 |
| Ratio fat mass/lean body mass | 0.38 ± 0.09 | 0.37 ± 0.09 | -0.01 ± 0.02 | | | 0.34 ± 0.10 | | | 0.33 ± 0.10 | -0.01 ± 0.02 | | | | **<0.001** | | **0.033** | 0.11 |
| **Strength** | | | | | | | | | | | | | | | | | |
| Grip strength, kg | 43 ± 7 | 43 ± 7 | 0 ± 4 | | | 46 ± 7 | | | 47 ± 8 | 1 ± 4 | | | | **<0.001** | | 0.07 | 0.52 |
| **Physical performance** | | | | | | | | | | | | | | | | | |
| SPPB total, pt | 12 (11 – 12) | 12 (11 – 12) | 0 (0 – 0) | | | 12 (11 – 12) | | | 12 (12 – 12) | 0 (0 – 0.3) | | | | **0.047** | | 0.55 | 0.63 |
| Balance, pt | 4 (4 – 4) | 4 (4 – 4) | 0 (0 – 0) | | | 4 (4 – 4) | | | 4 (4 – 4) | 0 (0 – 0) | | | | 0.99 | | 0.42 | 0.42 |
| Gait speed, pt | 4 (4 – 4) | 4 (4 – 4) | 0 (0 – 0) | | | 4 (4 – 4) | | | 4 (4 – 4) | 0 (0 – 0) | | | | **-** | | - | - |
| Gait speed, s | 3.2 ± 0.3 | 3.0 ± 0.4 | -0.2 ± 0.4 | | | 3.1 ± 0.4 | | | 2.9 ± 0.3 | -0.2 ± 0.5 | | | | **<0.001** | | 0.43 | 0.57 |
| Chair-rise, pt | 4 (3 – 4) | 4 (3 – 4) | 0 (0 – 0) | | | 4 (3 – 4) | | | 4 (4 – 4) | 0 (0 – 0) | | | | **0.038** | | 0.25 | 0.85 |
| Chair-rise, s ^a^ | 10.4 ± 2.2 | 9.7 ± 2.1 | -0.7 ± 2.2 | | | 10.3 ± 1.7 | | | 9.3 ± 2.0 | -1.0 ± 1.6 | | | | **<0.001** | | 0.59 | 0.52 |
| TUG, s | 7.0 ± 0.9 | 6.6 ± 0.8 | -0.4 ± 1.0 | | | 6.7 ± 0.9 | | | 6.2 ± 0.8 | -0.5 ± 0.7 | | | | **<0.001** | | 0.10 | 0.58 |
| Estimated VO_2_max, ml/kg/min ^b^ | 31.0 ± 10.2 | 38.9 ± 31.4 | 7.9 ± 31.2 | | | 30.4 ± 9.2 | | | 33.4 ± 11.4 | 3.0 ± 6.9 | | | | **<0.001** | | 0.61 | 0.77 |
| Data are presented as mean ± SD or median (interquartile range (IQR)).  MVC, maximal voluntary contraction; SPPB, Short Physical Performance Battery; TUG, Timed Up-and-Go; VO_2_max, maximal rate of oxygen consumption.  ^a^ *n =* 82, ^b^ Estimated VO_2_max, corrected for age and weight with the Åstrand test (*n =* 90).  Bold values indicate p-value < 0.05. | | | | | | | | | | | | | | | | | |

| **Online Resource 2B** Changes in body composition, strength, physical performance, blood and urine parameters of the female participants in the protein and placebo group | | | | | | | | | | | | | | | | | |
| --- | --- | --- | --- | --- | --- | --- | --- | --- | --- | --- | --- | --- | --- | --- | --- | --- | --- |
|  | **Protein** n=11 | | |  | | | | **Placebo** n=10 | | | | |  | | **P-value** | | |
|  | Pre | Post | Change | |  | | Pre | | Post | | Change |  | | | Time | Treatment | Interaction |
| **Body composition** | | | | | | | | | | | | | | | | | |
| Body weight, kg | 76.87 ± 11.53 | 75.57 ± 11.40 | -1.30 ± 1.22 | | | 68.20 ± 6.76 | | | 68.51 ± 6.90 | 0.31 ± 1.22 | | | | 0.08 | | 0.07 | **0.007** |
| Lean body mass, kg | 44.68 ± 5.18 | 45.23 ± 5.40 | 0.54 ± 1.34 | | | 40.06 ± 4.45 | | | 40.61 ± 4.15 | 0.54 ± 1.36 | | | | 0.08 | | **0.040** | 1.00 |
| Lean body mass, % | 58.58 ± 4.34 | 60.29 ± 4.28 | 1.70 ± 1.73 | | | 59.00 ± 5.25 | | | 59.52 ± 5.12 | 0.52 ± 1.99 | | | | **0.013** | | 0.93 | 0.16 |
| Fat mass, kg | 29.58 ± 7.03 | 27.8 ± 6.91 | -1.78 ± 1.43 | | | 25.72 ± 5.01 | | | 25.52 ± 4.90 | -0.20 ± 1.61 | | | | **0.007** | | 0.26 | **0.027** |
| Fat mass, % | 38.08 ± 4.58 | 36.37 ± 4.51 | -1.71 ± 1.63 | | | 37.56 ± 5.07 | | | 37.05 ± 5.01 | -0.50 ± 1.98 | | | | **0.011** | | 0.97 | 0.14 |
| Ratio fat mass/lean body mass | 0.62 ± 0.12 | 0.58 ± 0.11 | -0.04 ± 0.04 | | | 0.61 ± 0.13 | | | 0.60 ± 0.12 | -0.01 ± 0.05 | | | | **0.012** | | 0.95 | 0.15 |
| **Strength** | | | | | | | | | | | | | | | | | |
| Grip strength, kg | 30 ± 6 | 29 ± 6 | -1 ± 4 | | | 26 ± 7 | | | 26 ± 5 | 0 ± 2 | | | | **0.022** | | 0.24 | 0.34 |
| **Physical performance** | | | | | | | | | | | | | | | | | |
| SPPB total, pt | 11 (11 – 12) | 12 (11 – 12) | 0 (-1 – 1) | | | 12 (12 – 12) | | | 12 (12 – 12) | 0 (-1 – 0) | | | | 0.95 | | 0.55 | 0.20 |
| Balance, pt | 4 (4 – 4) | 4 (4 – 4) | 0 (0 – 0) | | | 4 (4 – 4) | | | 4 (4 – 4) | 0 (0 – 0) | | | | 0.96 | | 0.34 | 0.34 |
| Gait speed, pt | 4 (4 – 4) | 4 (4 – 4) | 0 (0 – 0) | | | 4 (4 – 4) | | | 4 (4 – 4) | 0 (0 – 0) | | | | **-** | | - | - |
| Gait speed, s | 3.3 ± 0.4 | 2.8 ± 0.5 | -0.5 ± 0.6 | | | 3.5 ± 0.6 | | | 3.3 ± 0.6 | -0.3 ± 0.3 | | | | **0.001** | | 0.09 | 0.25 |
| Chair-rise, pt | 4 (3 – 4) | 4 (4 – 4) | 0 (0 – 1) | | | 4 (3.8 – 4) | | | 4 (3.8 – 4) | 0 (-0.3 – 0) | | | | 0.96 | | 0.79 | 0.27 |
| Chair-rise, s ^a^ | 10.2 ± 2.0 | 9.4 ± 2.7 | -1.1 ± 2.0 | | | 9.7 ± 2.1 | | | 9.9 ± 3.2 | 0.6 ± 2.6 | | | | 0.63 | | 0.74 | 0.15 |
| TUG, s | 6.7 ± 0.6 | 6.4 ± 0.9 | -0.3 ± 0.5 | | | 7.9 ± 1.8 | | | 7.4 ± 1.7 | -0.4 ± 0.6 | | | | **0.004** | | 0.06 | 0.64 |
| Estimated VO_2_max, ml/kg/min ^b^ | 30.8 ± 8.9 | 35.0 ± 14.0 | 4.2 ± 10.7 | | | 25.7 ± 7.4 | | | 29 ± 4.8 | 3.3 ± 6.3 | | | | 0.07 | | 0.15 | 0.82 |
| Data are presented as mean ± SD or median (interquartile range (IQR)).  MVC, maximal voluntary contraction; SPPB, Short Physical Performance Battery; TUG, Timed Up-and-Go; VO_2_max, maximal rate of oxygen consumption.  ^a^ *n =* 19, ^b^ Estimated VO_2_max, corrected for age and weight with the Åstrand test (*n =* 21).  Bold values indicate p-value < 0.05. | | | | | | | | | | | | | | | | | |
